# Evolutionary Dynamics and Population Genetics of Ash Shoestring-Associated Virus in a European-Wide Survey

**DOI:** 10.3390/microorganisms13030633

**Published:** 2025-03-11

**Authors:** Sahar Nouri, Susanne von Bargen, Artemis Rumbou, Thomas R. Gaskin, Carmen Büttner, Shaheen Nourinejhad Zarghani

**Affiliations:** 1 Division of Phytomedicine, Faculty of Life Sciences, Albrecht Daniel Thaer-Institute of Agricultural and Horticultural Sciences, Humboldt University of Berlin, Lentzeallee 55–57, 14195 Berlin, Germany; sahar.nouri@hu-berlin.de (S.N.); susanne.von.bargen@agrar.hu-berlin.de (S.v.B.); carmen.buettner@agrar.hu-berlin.de (C.B.); nourines@hu-berlin.de (S.N.Z.); 2 Brandenburg State Office of Rural Development, Agriculture and Land Consolidation, Müllroser Chaussee 54, 15236 Frankfurt (Oder), Germany; thomas.gaskin@lelf.brandenburg.de

**Keywords:** emaravirus, forest, negative-strand RNA virus, evolution, population parameters, genetic differentiation

## Abstract

Ash shoestring-associated virus (ASaV; *Emaravirus fraxini*) is a five-segmented, negative-sense RNA virus associated with chlorosis and leaf deformation in *Fraxinus* species. This study investigated the genetic diversity and evolutionary dynamics of ASaV by analyzing nearly full-length RNA2–RNA5 and partial RNA1 sequences from isolates collected from different geographic regions and *Fraxinus* hosts. The sequence data uncovered that ASaV has a conserved genome, in which RNA3 and RNA5 showed more genetic divergence than other segments in the sequenced isolates. ASaV RNA3 and partial RNA1 were the most informative genomic regions for phylogenetic studies. There was a correlation between the clustering of the ASaV isolates and host species when the phylogenetic tree was constructed based on the RNA1 region. The ASaV genome is predominantly under purifying selection. Newly designed primers in this study facilitated robust amplification of genomic regions.

## 1. Introduction

Viruses are among the most widespread and abundant entities on Earth, playing a significant role in shaping ecosystems [1]. They are well recognized as major pathogens in crop plants, where their impacts on agriculture and food production have been extensively studied [2]. However, their role in woody plants, particularly forest trees, remains largely unexplored and is an emerging area of research [3]. Ash trees (*Fraxinus* spp., Oleaceae) are among European forests’ most ecologically and economically significant broadleaved species [4]. A recent survey revealed that ash trees are also affected by several plant viruses [5]. Among these, ash shoestring-associated virus (ASaV, *Emaravirus fraxini*) is a novel emaravirus, first identified in Switzerland in 2016 using next-generation sequencing (NGS) [6]. In *F. excelsior*, ASaV was the second most abundant virus after cytorhabdoviruses, with an incidence of 82% in trees showing virus-suspected leaf symptoms [5]. Infected trees exhibited symptoms such as leaf mottling, chlorosis, and abnormal growth patterns, including leaf curling and shoestring formation [6]. ASaV belongs to the genus *Emaravirus* within the order *Elliovirales* and the family *Fimoviridae* [7,8]. Virions of ASaV are enveloped harboring five negative-sense single-stranded RNAs with the conserved 13 nucleotide regions specific for emaraviruses at both termini of each genomic segment [8,9]. Each genomic fragment encodes a distinct protein: RNA-dependent RNA polymerase (RdRP, 268 KDa), glycoprotein precursor (GPP, 73 KDa), nucleocapsid protein (NP, 35 KDa), movement protein (MP, 41 KDa), and a 26 KDa protein with an unknown function (P5), encoded by RNA1 to RNA5, respectively [6]. As the fastest-evolving plant pathogens, viruses exhibit a high capacity for adaptation, enabling them to infect new host plants, overcome resistance, and alter symptoms and virulence [10,11].

Little is known about the genetic variability of ASaV. Understanding genome variability within viral populations is essential to developing more effective detection methods and strategies for viral disease control, such as searching for resistant ash varieties. Therefore, we investigated the genetic diversity of ASaV across different geographic regions and host plants by analyzing near full-length ASaV RNA2, RNA3, RNA4, and RNA5, as well as partial RNA1.

## 2. Materials and Methods

### 2.1. Sampling, RNA Extraction, and cDNA Synthesis

**Figure 1:**
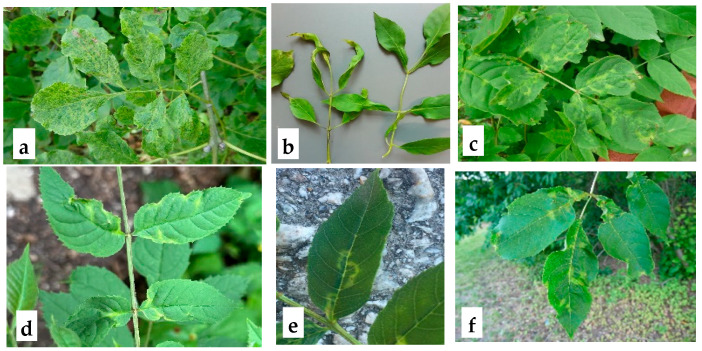
Symptoms observed in collected *Fraxinus* spp. leaf samples. **a**: Leaf deformation and mottling in *F. ornus* (Hamburg, Germany); **b**: shoestring symptoms in *F. excelsior*, characterized by elongated and narrowed leaves (Melzower forest, Germany); **c**: chlorotic line pattern and leaf deformation in *F. excelsior* (Sigtuna, Sweden); **d**: leaf deformation and line pattern in F. excelsior (Oberbozen, Italy); **e**: chlorotic line pattern on *F. excelsior* leaves (Basel, Switzerland); **f**: chlorosis and leaf deformation in *F. excelsior* (Bromma, Sweden). The presence of ASaV has been confirmed by RT-PCR.

The characteristics of the samples used in this study are presented in Supplementary Table S1. Several leaves showing ASaV symptoms (Figure 1) from a total of 51 trees (*F. ornus* and *F. excelsior*) from Germany, Sweden, Switzerland, and Italy were collected. The total RNA from the collected leaves was extracted following the protocol described by [12]. A total of 1.2 µg of extracted RNA was used as a template for cDNA synthesis using the Maxima H Minus Reverse Transcriptase (200 U/μL) kit (Thermo Fisher Scientific™, Waltham, MA, USA) with random hexamers in a 20 µL reaction volume, adhering to the manufacturer’s protocol.

### 2.2. PCR Amplification, Cloning, and Sequencing

The sequence of the ASaV isolate (GenBank accession number (acc. no.) OU466880−84) was used for designing the primer sets targeting near full-length segments of RNA2 to RNA5, including the ORF2 to ORF5 and flanking untranslated regions. The PCR products for RNA2–5 covered almost the full length of the corresponding genomic RNAs: RNA2 (2225 out of 2243 nt in the reference isolate, acc. no. OU466881), RNA3 (1473 out of 1483 nt in acc. no. OU466882), RNA4 (1510 out of 1518 nt in acc. no. OU466883), and RNA5 (1330 out of 1333 nt in the reference isolate, acc. no. OU466884). Primer sets targeting two regions of ORF1 on the RNA1—hereafter be referred to as the ORF1 N-proximal region (antigenome positions 840–1875 nt, acc. no. OU466880) and the ORF1 C-proximal region (antigenome positions 4362–5470 nt, acc. no. OU466880)—were designed using the sequences of ASaV isolates (acc. nos. OU466880 and OU46687) (Table 1). PCR amplification was performed using Phusion DNA Polymerase (2 U/µL) (Thermo Fisher Scientific™, Waltham, MA, USA), a high-fidelity enzyme with proofreading 3′ → 5′ exonuclease activity, in a 25 μL reaction following the manufacturer’s instructions. The RT-PCR results are presented in Supplementary Figures S1–S6. Amplified RT–PCR products were ligated into the pJET1.2 blunt-end cloning vector (CloneJET PCR Cloning Kit, Thermo Fisher Scientific™, Waltham, MA, USA) and transferred into *Escherichia coli* XL1-Blue MRF competent cells (Stratagene; Thermo Fisher Scientific™, Waltham, MA, USA). Colony PCR using pJET1.2F and pJET1.2R primers (provided by the kit) was applied to select the recombinant plasmids. Among the screened colonies harboring the recombinant plasmid with the expected insert, at least two and a maximum of six independent colonies were subjected to plasmid extraction using the NucleoSpin Plasmid EasyPure kit (Machery Nagel, Düren, Germany) and were sent for sanger sequencing to Macrogen Europe (Amsterdam, The Netherlands) using vector-specific primers (pJET1.2F and pJET1.2R) and gene-specific primers to cover the gaps (Table 1).

### 2.3. Sequence Data Analysis, Recombination, Phylogeny, and Population Parameters

The obtained sequence data were assembled using the BioEdit Sequence Alignment Editor version 7.2.5 [13] and deposited in GenBank with acc. no. PQ438405 to PQ438536. The assembled sequences were aligned using the MUSCLE algorithm, implemented in MEGA11 [14].

Seven recombination detection methods (RDP, BOOTSCAN, GENECONV, MAXCHI SISCAN, 3SEQ, and LARD) implemented in the RDP4 software were applied to detect recombination events in the aligned sequences [15]. Recombination events identified by less than four independent methods were considered unreliable. Phylogenetic trees were constructed using the neighbor-joining (NJ) and maximum likelihood (ML) methods with bootstrap analysis in MEGA11 software to investigate the evolutionary relationships among the sequence isolates of the virus. It should be mentioned that the branching patterns of the NJ and ML trees were essentially similar; however, the NJ trees are shown in this paper. Branches with bootstrap values lower than 90% were collapsed. Corresponding regions of *Emaravirus kiwii* (acc. no. NC_055646, NC_055647, NC_055648, NC_055649, and NC_055651), *Emaravirus fici* (acc. no. PP196567, PP196569, PP228020, PP228021, and PP228022), and *Emarovirus toordali* (acc. no. HF912243, HF912244, HF912245, HF912246, and HG939490) were used as outgroups for phylogenetic trees. For the phylogenetic analyses of ASaV RNA5, the RNA6 of *Emaravirus fici* (PP196569), *Emaravirus toordali* (HG939490), and *Emaravirus kiwii* (NC_055651), were used as an outgroup as the P6 of these emaraviruses share more homologous motifs with ASaV P5 [6]. SignalP 6.0 (https://services.healthtech.dtu.dk/services/SignalP-6.0/ (accessed on 10 July 2024)) and TMHMM v2.0 (https://services.healthtech.dtu.dk/services/TMHMM-2.0/ (accessed on 10 July 2024)) were used to predict the presence and characteristics of signal peptides and transmembrane helices. NetNGlyc-1.0 (https://services.healthtech.dtu.dk/services/NetNGlyc-1.0/ (accessed on 10 July 2024)) was used to predict N-glycosylation sites in the ASaV isolates’ GPP sequences. DnaSP version 6.12.03 software [16] was used to assess different population parameters.

## 3. Results

### 3.1. Nucleotide Identity Comparisons

The two regions of the ASaV RNA1 genome, the ORF1 N-proximal region and ORF1 C-proximal region, are located at positions 840–1875 and 4361–5474 (numbers based on antigenome reference isolate acc. no. OU466880), respectively. The pairwise comparisons of 11 samples including E59464, E60765, E62017, E62033, E62787, E62907, E63064, E63065, E63069, E63071, and E63073 for ORF1 N-proximal and E60765, E62017, E62033, E62907, E63064, E63065, E63069, E63071, E63073, E62477, and E62941 for ORF1 C-proximal, along with two isolates from GenBank (accession numbers OU466880 and OU466875), showed nucleotide-level identities of 92.88–100% and 87.62–99.91%, respectively, in the sequenced isolates. In the ORF1 N-proximal region, the highest identity (100%) was recorded between E62907 (Melzower forest in eastern Germany) and isolates E63069 and E63073 (from Jever and Wangerland in the west of Germany). The lowest identity (92.88%) was observed between E62033, sampled from Melzower forest in northeast Germany, and E55270, sampled from Basel, Switzerland. In the ORF1 C-proximal region, the lowest identity (87.62%) was observed between isolate S10 (Hamburg, Germany) and E63064 (Wangerland, Germany). The highest identity (99.91%) was observed between two isolates from Melzower, forest E62477 and E62033. Interestingly, these isolates shared only 89% identity with another isolate from the same location, E62907, indicating genetic variability among the group of isolates collected from the same location (Melzower forest, Germany).

RNA2 was amplified in 20 isolates, RNA3 in 41 isolates, RNA4 in 26 isolates, and RNA5 in 23 isolates. These sequences were used for pairwise alignment comparisons with available ASaV genome sequences in the GenBank database, including RNA1–RNA5. The GenBank isolates used for alignment included RNA2 (OU466881), RNA3 (MN399721, MN399722, OP501825, OU466877, OU466872, OU466882), RNA4 (OP501826, OU466878, OU466883), and RNA5 (OU466879, OU466884). The nucleotide identity of 92.75–100%, 91.97–100%, 94–100%, and 90–100% was obtained in pairwise comparisons of almost full-length RNA2 to RNA5 of the sequenced isolated (Supplementary Table S2). RNA4 was the most conserved genomic fragment, while RNA5 and RNA3 were the most divergent genomic segments of ASaV in the studied isolates. The E62015 (Melzower forest, Germany) isolate was the most divergent isolate based on the RNA2. Based on the pairwise sequence identity of RNA3, the lowest identity was observed between E62779 (Kaisheim, Germany) and E62017 (Berlin, Germany). The amino acid’s lowest identity (93.1%) was observed between E58051 (*F. ornus*, Hamburg, Germany) and a group of isolates including E62017 (Berlin, Germany), E62037, E62053, E62073, E62900 (Melzower forest, Germany), and PaEV (*Pisum sativum*, Meissen county, Germany, acc. no. MN399722). In the case of RNA4, the lowest sequence identity (95%) was observed between isolates E62900 (Melzower forest, Germany) and OP501826.1 (Castets-en-Dorthe, France). At the deduced amino acid (aa) level, the sequence identity exceeded 99.16%. The lowest observed identity based on the RNA5 was found between isolates E62001 (Hamburg, Germany) and E62231 (*F. ornus*, Berlin, Germany).

The nucleotide comparisons between the isolates collected from the same location based on the sampling sites, i.e., Berlin, Melzower forest, Hamburg, Wangerland, Jever, Wittmund, Goting, and Kaisheim, showed that the isolates collected from Berlin and Melzower forest had the most divergent isolates.

### 3.2. Recombination and Phylogenetic Studies

**Figure 2:**
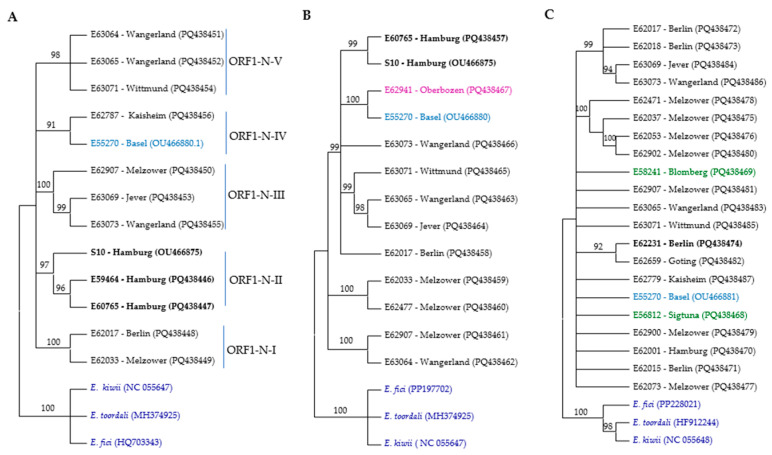

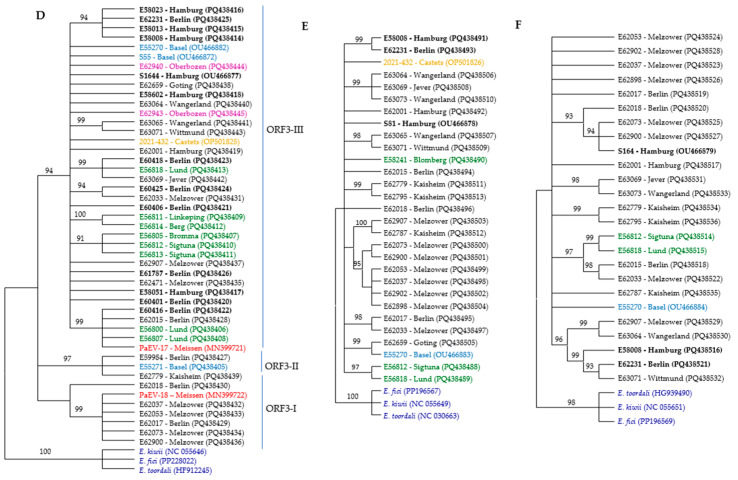
Phylogenetic trees of RNA segments from different ASaV isolates based on nucleic acid were constructed using the neighbor-joining method with 1000 bootstrap replications in MEGA11. The trees represent (**A**) ORF1 N-proximal; (**B**) ORF1 C-proximal; (**C**) RNA2; (**D**) ORF3; (**E**) RNA4; and (**F**) RNA5. The color scheme indicates the geographic origin of the isolates: blue for Swiss isolates, black for German isolates, red for PaEV, brown for French isolate, green for Swedish isolates, lilac for Italian isolates, and dark blue for outgroups. Bold labels correspond to *F. ornus*. Branches with bootstrap values below 90% were collapsed.

There were no differences between the ML and NJ trees; therefore, only the NJ trees are presented (Figure 2). The phylogenetic trees constructed using the amino acid sequences of partial ORFs (in the case of ORF1) or complete ORF sequences were not phylogenetically informative, except for ORF3 (Figure 2D). In addition, the phylogenetic trees constructed using the nucleotide sequences of RNA2 and RNA5 were not phylogenetically informative, as most isolates remained ungrouped. Therefore, only the phylogenetically informative trees have been described and analyzed in this study. There was no correlation between the geographical origin of the isolates and phylogenetic groups. Furthermore, there was no correlation between the host species and the phylogenetic trees based on RNA2–RNA5. However, isolates collected from *F. ornus* formed a distinct cluster in the tree constructed using the partial ORF1 sequence.

For constructing the phylogenetic trees based on the ORF1 N-proximal and ORF1 C-proximal regions, we successfully amplified these regions in 11 samples, 9 of which originated from the same source. Specifically, for isolates E62787 and E59984, only the ORF1 N-proximal region was successfully amplified, whereas for isolates E62941 and E62477, only the ORF1 C-proximal region was successfully amplified. Based on the ORF1 N-proximal, ASaV isolates clustered in five groups (Figure 2A). Isolates E62017 and E62033 clustered in the clade ORF1-N-I; isolates S10, E59464, and E60765 in clade ORF1-N-II; isolates E62787 and E55270 in clade ORF1-N-III; isolates E62907, E63069, and E63073 in clade ORF1-N-IV; and isolates E63064, E63065, and E63071 in clade ORF1-N-V. This profile differed when the NJ tree was constructed based on the ORF1 C-proximal. The ASaV isolates were grouped in three main clusters (Figure 2B), and there was no consistency in profiling with the NJ tree constructed based on the ORF1 N-proximal. This observation may suggest the occurrence of a recombination event within RNA1.

A phylogenetic analysis based on near full-length nucleotide sequences of ORF2 identified that five clades and six isolates (34%) remained ungrouped (Figure 2C).

Three different NJ trees were constructed based on the near full-length nucleotide sequences of ASaV RNA3 in sequenced isolates and the ORF3 region at both the nucleotide and amino acid levels. ASaV isolates consistently grouped into three main clades across all constructed trees, except for isolate E59984, which remained ungrouped in the amino acid-level tree and did not cluster with its expected clade, ORF3-II. In addition, also using additional ASaV ORF3 sequences that were available in GenBank, a constructed tree based on the nt sequence of ORF3 is presented (Figure 2D). Based on the latter tree, clade ORF3-I comprises six ASaV isolates; E62017 and E62018, both from Berlin (Germany); isolates E62037, E62053, E62073, and E62900 from Melzower forest (Germany); and the PaEV-18 isolate reported from the pea (*Pisum sativum*, Meissen county, Germany, acc. no. MN399722). Clade ORF3-II includes three isolates, E62779 (Kaisheim, Germany), E55271 (Basel, Switzerland), and E59984 (Berlin, Germany), which all share the same host species, *F. excelsior*. The remaining isolates placed in clade ORF3-III originated from various geographical regions and were isolated from different host species. As mentioned, there was no correlation between the geographical or host origins and phylogenetic groups.

Nearly the full length of RNA4 was amplified in 26 isolates and used for the construction of the NJ tree, and three reference sequences were retrieved from GenBank: OP501826 (*F. excelsior*, Castets-en-Dorthe, France); OU466878, S81 (*F. ornus*, Hamburg, Germany); and OU466883, E55270 (*F. excelsior*, Switzerland). The ASaV isolates were grouped in eight distinct clades, and six isolates (almost 20% of isolates) remained ungroup (Figure 2E). A similar clustering profile was obtained when the NJ tree was constructed based on the nt sequences of ORF 4 (Supplementary Figure S7).

The ASaV isolates were categorized in five different clades, and seven isolates (almost 28% of isolates) remained ungrouped when the NJ tree was constructed based on the nucleotide sequence of ASaV RNA5 (Figure 2F).

The RNA2–RNA5 sequences of the ASaV isolates were analyzed for recombination events. No recombination sites were found in any of the segments, as confirmed by four independent methods.

### 3.3. Indel Events and Motifs in Different Genomic RNAs of ASaV

The alignment of RNA2 from 21 ASaV isolates revealed that all sequences terminate with a UAA stop codon, differing from the GenBank-retrieved E55270 isolate (OU466881), which terminates with a UAG stop codon. A putative cleavage site at KA ↓ DD [17], located between A_182_ and D_183_, was identified in all isolates analyzed. This site is predicted to generate a smaller 21 kDa glycoprotein (Gn) and a larger 51 kDa glycoprotein (Gc). The Pi_a_/Pi_s_ values were calculated as 0.063 for Gn and 0.034 for Gc region of ASaV GPP. The GCYDCQSG motif [17] was conserved across all ASaV isolates at positions 475–482. The predicted N-glycosylation sites at four positions (N_62_, N_328_, N_390_, and N_623_) with high confidence were also conserved across all isolates. SignalP-5.0 predicts a signal peptide with the cleavage site occurring at positions 22–23 in the VYN–HF context with a 55.07% probability. Four transmembrane helix (TMH) domains were identified between residues 2–24, 112–134, 170–187, and 581–603.

The conserved sequence motifs NXXSXNXXXA, NRLA, and GYFE, believed to be involved in RNA binding within the NP of emaraviruses [18], were consistently present across all studied isolates. These motifs correspond to the sequences NIVSFNKACA at positions 130–139, NRLA at 178–181, and GYFE at 192–202 nt. The isolates belonging to clade ORF3-I exhibit distinguishing features that differentiate them from those in clades ORF3-II and ORF3-III. Protein synthesis in these isolates terminates at an opal (UGA) stop codon, and clade ORF3-II and ORF3-III isolates terminate at an ochre (UAA) stop codon. Two PaEV isolates retrieved from GenBank with two different stop codons (PaEV-17 acc no. MN399721 with UAA and PaEV-18 acc. no. MN399722 with UGA) were classified as clades ORF3-III and ORF3-I. Interestingly, shorter RNA3 lengths, unique amino acid substitutions, and different stop codons were observed in the ASaV RNA3 clade ORF3-I isolates.

Alignment of the RNA4 sequences confirmed that the signal-peptide sequence at both the P4 N-terminus and the C-terminus, along with motifs critical for cell-to-cell movement (K_55_, Y_72_, R_88_XA_90_XXXXXW_96_XP_98_, D_113_XR_115_, V_146_, D_168_I_169_XK_171_I_172_, M_183_, W_197_XT_199_, F_215_, E_224_, and I_326_ [19,20,21]), were conserved in all studied isolates. All studied isolates terminated with a UAG stop codon (antigenome position 1169–1172 nt). Within this region, the ORF4 gene consists of 1086 nucleotides and encodes a protein of 362 amino acids, corresponding to antigenome positions 83 to 1168 in the reference sequence OU466883. An indel event was identified at antigenome position 55 (Adenine) of the 5′-UTR, resulting in a variable length of 49–50 nucleotides (antigenome positions 34–84; accession no. OU466883). The alignment of the 3′-UTR, encompassing 330 out of 350 nucleotides (antigenome positions 1168–1518; accession no. OU466883), revealed that indels occur in the AT-rich region, resulting in varying lengths of 324 to 330 nucleotides. Due to indels in this region, E62907 was the longest, while E62037, E62053, and E62902 (Melzower forest, Germany) were the shortest. Interestingly, this deletion occurred in the isolates, including five out of eight from Melzower forest and one isolate (E62017) out of four from Berlin.

As with RNA3 and RNA4, the isolates studied for RNA5 exhibited variations in sequence lengths due to indels in the 3′-UTR. The RNA5 segment length of the isolates ranges between 1277 and 1288 nucleotides, compared to the full RNA5 length of 1333 nucleotides (antigenome positions 28–1302, acc. no. OU466884). The ORF5 consists of 693 nucleotides (antigenome positions 70–762, acc. no. OU466884), encoding a protein of 231 amino acids. All studied isolates terminated with a UAA stop codon.

### 3.4. Analysis of Population Parameters

The estimated nucleotide diversity parameters for different genomic RNAs of ASaV revealed that the ORF1 C-proximal showed a higher value of nucleotide diversity (*π* = 0.05957 ± 0.00775) and Watterson’s Theta (θ*_W_* = 0.05758 ± 0.02187) in comparison to the other genomic RNAs, followed closely by RNA5 (π = 0.05552 ± 0.00318; θ*_W_* = 0.05183 ± 0.01678). RNA3 showed moderate to high nucleotide diversity, and RNA4 showed the lowest nucleotide diversity. The haplotype diversity ranged from 0.03282 ± 0.01034 to 0.05758 ± 0.02187 for all genomic RNA segments, denoting moderate haplotype diversity.

An estimation of the ratio of nonsynonymous substitutions to synonymous substitutions (Pi_a_/Pi_s_) or selection pressure on different genomic regions of the ASaV genome revealed that all studied regions are under strong purifying selection pressure (Pi_a_/Pi_s_ ranged from 0.00874 to 0.0417) (Table 2). However, this pressure was not distributed equally across different genomic RNAs or within different regions of the same genomic RNA.

The tree based on the deduced amino acids of the ORF1 N-proximal forms two clades (Supplementary Figure S8). Clade II contains two isolates, E62033 (Melzower forest, Germany) and E62017 (Berlin, Germany). The conserved amino acids in these two isolates (S_308_, L_347_, T_389_, I_416_, I_426_, and K_494_) differ from those in all isolates of clade I, where the corresponding residues are A_308_, S_347_, A_389_, V_416_, L_426_, and M_494_. A site-specific selection pressure analysis indicated that L_347_ was under positive selection, and other residues were under negative selection.

Multiple alignments of deduced amino acid sequences of ORF3 revealed seven conserved residues (G_9_, A_13_, T_18_, I_41_, M_109_, E_157_, and K_163_) among all isolates belonging to clade ORF3-I. At the same sites, the conserved amino acids in the clade ORF3-III isolates are T_9_, V_13_, A_18_, V_41_, L_109_, K_157_, and A_163_, and in the clade ORF3-II isolates, by a slight difference of clade ORF3-III, are S_9_, V_13_, A_18_, V_41_, L_109_, K_157_, and E_163_. A site-specific selection pressure analysis indicated that G_9_ is under positive selection, A_13_ and T_18_ are under negative selection, and the other residues are under neutral selection.

Tajima’s *D*, Fu and Li’s D*, and Fu and Li’s F* test values were negative for RNA2 to RNA4, indicating an excess of rare alleles, consistent with population expansion, selective sweeps, or bottlenecks. In contrast, the studied partial regions of ORF1 and RNA5 showed positive Tajima’s *D* values, suggesting balancing selection or population bottlenecks. Additionally, Fu and Li’s D* and Fu and Li’s F* values for these regions (0.10779 and 0.19384, respectively) were close to neutral, providing weak evidence of selection.

*F_ST_* was used to determine whether certain clades ORF3-I, II, and III were differentiated. The results are presented in Table 3 and were evaluated using the statistical tests *K*_s_*, Z*, and Snn, which provided significant values, suggesting strong genetic differentiation among the phylogroups. *F*_ST_ values greater than 0.620 indicate that limited genetic exchange between the RNA3 phylogroups may have occurred.

Population differentiation tests were conducted for the three phylogenetic groups identified in the NJ tree constructed based on RNA3. The results showed that all *K*_s_*, Z*, and Snn values were statistically significant, indicating genetic differentiation among these phylogroups. The *F_ST_* values provide an additional measure of genetic differentiation, and higher *F_ST_* values indicate strong genetic structure, with limited gene flow among the clades.

## 4. Discussion

The expanding catalog of emaraviruses, including new members and sequenced isolates, has enriched our understanding of their genome structure, detection methods, diversity, evolution, and host adaptation. Advances such as high-throughput sequencing have facilitated the identification of conserved motifs (e.g., motifs A–E) in the RNA-dependent RNA polymerase (RdRP) of emaraviruses, enabling the design of genus-specific primers for virus detection [22]. Additionally, the pDAP13 primer, which targets a conserved 13-nucleotide sequence at both the 5′ and 3′ ends of all emaravirus genomic RNAs, has been widely used to amplify entire genomes [23]. However, its efficiency varies by genomic region, necessitating supplementary primers for precise amplification.

In this study, we analyzed the genetic diversity of ASaV using two regions of RNA1 and near-complete sequences of RNA2–RNA5 from isolates collected across two *Fraxinus* species and diverse geographic locations. At the time of primer design in 2022, only one complete ASaV genome in GenBank (E55270, Basel, Switzerland, *F. excelsior*, acc. no. OU466880–84) covering RNA1–5 and one fully sequenced RNA1 genome (S10, Germany, Hamburg, *F. ornus*, acc. no. OU466875) were available from high-throughput sequencing. Screening with primers from previous studies [6] amplified short fragments (243–500 bp), which were not sufficient for the genetic diversity studies, but newly designed primers (Table 1) demonstrated robust amplification across all genomic regions, particularly RNA3. However, primers for the ORF1 N- and C-proximal showed lower efficiency, leading to a lower number of successfully amplified RNA1 fragments. This could be addressed by utilizing additional full-length RNA1 sequences in future studies. The pDAP13 primer was effective for RNA3–RNA5 but required downstream gel purification for separation of the RNA5 and cloning to distinguish RNA3 and RNA4 due to their similar lengths. This highlights the practical advantage of using region-specific primers like those developed here for genomic studies.

While the majority of genetic diversity studies on emaraviruses rely on short fragments from different genomic regions [6,18,21,24,25,26,27,28,29,30,31], some studies have successfully utilized larger genomic segments and a greater number of isolates [21,32,33,34,35]. A significant focus has been placed on the core genomic RNA molecules, RNA1–RNA4, which are present in all emaraviruses. Among these, RNA3, encoding the nucleocapsid protein (NP), has been identified as a promising candidate for genetic diversity studies due to its critical role in the virus life cycle and its variability [21]. Other RNA molecules, such as RNA1, RNA2, and RNA4, have also demonstrated different levels of variability but are less explored in genetic diversity studies.

It has been reported that the aspen mosaic-associated virus (AsMaV) RdRP N-terminus showed 85.2–99.7 identity, which was similar to the level of the AsMaV RNA3 identity (86.8–100%) at the nt level and the phylogenetic tree based on this region showed similar profiling [21]. The ASaV ORF1 N-proximal and C-proximal showed 92.88–100% and 87.62–99.91% identity in a pairwise comparison of the sequenced isolates while the same value for RNA3 was 91.97–100%. In addition, only phylogenetic trees based on both ORF1 N-proximal and C-proximal clustered the isolates based on their host species (Figure 2). This evidence raises the question of whether RdRP (ORF1) is a better candidate for genetic diversity and phylogenetic studies of the emaraviruses compared to NP. Similar findings have been reported for the potato leafroll virus, which has a very conserved genome, and P0 was a better candidate in comparison to the coat protein for clustering the isolates [36]. More sequence isolates from different species of emaraviruses are needed to evaluate this idea.

ASaV RNA3 and ASaV ORF3, which encode the nucleoproteins in all sequenced isolates, showed at least 91.97% and 90.93% identity at the nt level, despite differences in host plant origins and geographic distribution. As with other emaraviruses, the RNA3 identity varies across species. Blackberry leaf mottle-associated virus (BLMaV) (based on ORF3), rose rosette virus (RRV) (based on ORF3), redbud yellow ringspot-associated virus (RYRSaV) (based on ORF3), and jujube yellow mottle-associated virus (JYMaV) (based on partial RNA3: 885 nt) exhibit the highest NP identity, reported to be 95% to 100% [18,28,30,32]. The RNA3 identity in pear chlorotic leaf spot-associated virus (PCLSaV) (based on partial RNA3: 725 bp) isolates has a range from 91% to 99.9% [32]. In comparison, ASMaV (based on near full-length), and pigeonpea sterility mosaic virus 1 (PPSMV-1) and 2 (PPSMV-2) (based on partial RNA3: 947 bp and 995 bp) exhibit a sequence identity among isolates of 86.8% to 100% [21,28].

All ASaV isolates could be categorized based on RNA3 and ORF3, making this genomic region a good candidate for phylogenetic analysis beside the ORF1 N-proximal. A phylogenetic analysis of RNA3 revealed three distinct clades, indicating significant genetic divergence despite the absence of host or geographic structuring. This divergence was supported by high *F_ST_* values (> 0.6) and elevated synonymous substitution rates (*K*_s_* = 2.17–2.99). Both the Snn and Z* statistics were significant (*p* < 0.01), highlighting strong genetic differentiation among clades, likely shaped by historical population bottlenecks, founder events, or selective pressures. While the lack of host specificity suggests free viral movement among *Fraxinus* spp. trees, occasional long-distance dispersal may contribute to the observed geographic distribution of RNA3 clades. Notably, ASaV has only been detected in *Fraxinus* spp., with an unconfirmed report from *Pisum sativum* [37], leaving its potential host range uncertain.

Our findings also revealed that ASaV RNA3 and RNA5 were more divergent than other genomic RNA molecules (Table 2). These differences likely reflect distinct functional and selective constraints across the genome. The high identity in RNA2 and RNA4 suggests that these regions were under stronger evolutionary pressure to maintain essential functions. In contrast, the variability in RNA3 and RNA5 suggests their roles in viral adaptation to diverse host and environmental conditions. RNA3, which encodes the nucleocapsid protein, underscores its significance in diversity studies, with its variability potentially facilitating adaptation to different hosts. RNA5, showing higher genetic variability in comparison to the other genomic regions, may be involved in host interactions or regulatory functions, further highlighting its importance in viral evolution.

RNA2 showed at least 92.75% identity, and it was under purifying selection (Table 2), which can explain the lower genetic diversity of RNA2. The RNA2 of emaraviruses is known to encode glycoprotein precursors (GPPs), which are predicted to contain N-terminal signal peptides. In all ASaV isolates, the putative cleavage site is located at residues 22–23, as also observed in ASMaV and PPSMV-1. Four N-glycosylation sites and four transmembrane helix (TMH) regions were predicted in all ASaV isolates, though the number of such sites and regions varies among different emaraviruses [19,21].

ASaV isolates displayed differences in stop codons, which were also detected in the ORF3 region of the ASMaV and RYRSaV isolates [21,27]. The type of stop codon can affect non-canonical translation mechanisms in RNA viruses and play a regulatory role in modulating the amount of protein translation [38,39]. However, the impact of stop codon variation on ASaV RNA3 remains unclear. There was an indel of 3 or 5 nt in ORF3-I isolates, which also occurred in ASaV RNA4 and RNA5, as the 3′-UTRs of these segments also consist of repetitive adenine and uracil sequences. These events are more likely to occur in homopolymeric regions where repetitive nucleotide sequences heighten the chances of replication machinery slippage [40]. A variation in the length of RNA3 has also been reported in other emaravirus species, such as European mountain ash ringspot-associated virus (EMARaV), Wheat mosaic virus (WMoV), and AsMaV [21,41].

ASaV RNA2, RNA4, and RNA5 were not phylogenetically informative. Similar findings have been reported for other members of emaraviruses such as AsMaV, RRV, and PCLSaV [21,31,35]. The ASaV MP sequence was the most conserved gene among the ASaV genome segments studied. This is not the case in all emaraviruses; for example, the identity of the MP gene in the blackberry leaf mottle-associated virus (BLMaV) ranged from 86–99% at the nt level and 95–100% at the aa level [33].

Overall, a maximum divergence of 0.14 was observed among the ASaV isolates. However, most genomic regions of the ASaV genome exhibited more than 90% identity at the nucleotide level, with even higher identity observed in the coding regions and at the amino acid level. The nucleotide diversity ranged between 0.02700 ± 0.00126 and 0.05957 ± 0.00775, denoting that ASaV overall has a conserved genome. ASaV in comparison to other emaravirus members showed less nucleotide diversity, for example, the N terminal regions of RdRP (0.40), MP (0.49), and NP (0.21). The nucleotide diversity of different genomic regions ranged from 0.049 to 0.077 for AsMaV and from 0.022 to 0.043 for FMV NP [21,26]. This range of nucleotide diversity was in a similar range for some other woody plant-invading viruses, such as citrus tristeza virus (0.038) and citrus leaf blotch virus (0.021) [24,26,41] but was very low in comparison to grapevine fanleaf virus (0.15) [42]. It has been reported that the nucleotide diversity of blackberry leaf mottle-associated virus (BLMaV) was rather low when compared to FMV and European mountain ash ringspot-associated virus (EMARaV) [30,43], ranging from 0.034 to 0.057 for NP and MP genes, respectively. The ASaV NP and MP genes showed similar or even lower nucleotide diversity, 0.037 and 0.027, for the corresponding regions.

This could be explained by the very strong purifying selection pressure on the ASaV genome (Table 2). Negative values of Tajima’s *D*, Fu and Li’s D*, and Fu and Li’s F* were obtained for RNA2–4, suggesting an excess of low-frequency polymorphisms. This pattern may be the result of demographic events, such as population expansion or background selection [44], where deleterious alleles are removed through purifying selection. In contrast, positive values of Tajima’s *D*, Fu and Li’s D*, and Fu and Li’s F* in RNA1 (ORF1 C-proximal) and RNA5 suggest that variation in these genomic regions deviates from neutrality, with an excess of intermediate-frequency alleles. This may be driven by balancing selection or a population bottleneck [45].

In the viruses with segmented genomes, reassortment is also an important factor in the genetic diversity and evolution of these viruses. Placing different genomic RNAs of ASaV in different phylogenetic groups indicated the possibility of recombination or reassortment (Figure 2). However, no significant evidence of recombination was detected, which could be attributed to the limited availability of sequences, particularly from different regions, or the involvement of other factors such as reassortment or genetic drift in the evolution of these isolates. Recombination and reassortment play important roles in the diversity of HPWMoV [41]. Moreover, it has been reported that some FMV strains may have emerged through reassortments [26].

## 5. Conclusions

Taken together, ASaV exhibits a conserved genome structure characteristic of emaraviruses, with isolates grouped into distinct phylogroups based on RNA3 and the ORF1 N-proximal. While a phylogenetic analysis revealed no correlation with geographical origin or host speciation, certain phylogroups displayed unique indels and conserved amino acid residues, indicating localized adaptation or evolutionary pressures.

ASaV evolution appears predominantly shaped by purifying selection, as evidenced by negative Tajima’s *D* values for RNA2–RNA4 and Pi_a_/Pi_s_ ratios below 1. However, positive Tajima’s *D* values for the ORF1 C-proximal and RNA5 suggest the influence of balancing selection or demographic processes, such as population bottlenecks or gene flow. These conflicting signals can be explained by purifying selection acting on non-synonymous sites alongside neutral or demographic factors.

Demographic effects, such as population bottlenecks, founder events, or genetic drift, likely contributed to the observed patterns. Slightly positive neutrality indices (e.g., Fu and Li’s D* and F*) may also reflect the balancing selection on regulatory regions or synonymous sites, mutation-drift equilibrium in stable populations, or human-mediated dispersal of infected material. These findings underscore the complex interplay between selective and demographic forces in shaping ASaV evolutionary trajectory.

Moreover, this study demonstrates the utility of region-specific primers designed to target conserved and variable regions across the ASaV genome, which can significantly enhance virus detection capabilities. The lack of geographic or host-specific structuring among isolates further underscores the potential for long-distance viral dispersal and the need for broader surveillance across diverse environments.

## Figures and Tables

**Table 1:** Primers used to amplify near full-length regions of RNA2–5 and two partial regions of RNA1 in ASaV, including primers used for sequencing and detection.

Primer Name	Primer Sequence (5′ → 3′)	Genome Segment	Primer Position (5′ → 3′) *	Used For	PCR Product Size (bp)	Reference
ASaV-R1-840-s	AGCAGTCTATTCGGGGACAA	RNA1	840–859	cloning	1035	this study
ASaV-R1-1875-as	GTGTGAAGTATCTTAATGGAGCAG	RNA1	1875–1852			
ASaV-R1-4361-s	GCTTGGTAGATTAGATAGGC	RNA1	4361–4380	cloning	1113	this study
ASaV-R1-5474-as	CATGATGATGGAAGATGGTGATG	RNA1	5474–5452			
ASaV-R2-16-s	ATAATAAAGCCAATCAGCATAAAATC	RNA2	16–41	cloning	2225	this study
ASaV-R2-end-as	TAGTGAACTCCTCATAAGTACAAC	RNA2	2241–2218			
ASaV-R3-10-s	CTCCCATAACTATAAATCAGTCAAAAG	RNA3	10–36	cloning	~1473	this study
ASaV-R3-end-as	AGTAGTGAACTCCCATATCTTACAATC	RNA3	1483–1457			
ASaV-R4-10-s	CTCCTTACAACATAAGCAATCTGC	RNA4	10–33	cloning	~1510	this study
ASaV-R4-end-as	GTAGTGAACTCCTTACAAATGAAATC	RNA4	1517–1492			
ASaV-R5-1-s	AGTAGTGAACTCCCTATAAACAAWATC	RNA5	1–23	cloning	~1330	this study
ASaV-R5-end-as	TAGTGAACTCCCTATAACTAAATCAG	RNA5	1331–1306			
ASaV-R2-806-s	GATTACAAATGCTGTATATAGC	RNA2	806–827	sequencing	N/A **	this study
ASaV-R2-1366-as	ACATTATTGTCATATACTGATTCC	RNA2	1366–1343			
S55-579F	AAGATCTGCTCCTGATCCTGC	RNA3	607–627	detection	342	[6]
S55-920R	CTGGTTGTCCCAATATCTCTGG	RNA3	948–927			

* The primer positions are numbered according to the antigenome nucleotide reference isolate (acc. no. OU466880 for RNA1, OU466881 for RNA2, OU466882 for RNA3, OU466883 for RNA4, and OU466884 for RNA5). ** Not applicable.

**Table 2:** Neutrality tests and nucleotide diversity of ASaV isolates based on each genome RNA segments.

Genome Segment	n	S	θ*_W_*	π	Hd	Pi_a_	Pi_s_	Pi_a_/Pi_s_	Tajima’s *D*	Fu and Li’s D*	Fu and Li’s F*
**ORF1 N-proximal**	13	113	0.03678 ± 0.01412	0.03524 ± 0.00406	0.03678 ± 0.01412	0.00570	0.14870	0.03833	−0.19157	0.48894	0.34979
**ORF1 C-proximal**	13	191	0.05758 ± 0.02187	0.05957 ± 0.00775	0.05758 ± 0.02187	0.00548	0.25733	0.0213	0.15884	0.73035	0.65990
**RNA2**	21	368	0.04701 ± 0.01575	0.03564 ± 0.00414	0.04701 ± 0.01575	0.00614	0.14715	0.0417	−0.99952	−1.29441	−1.40857
**RNA3**	42	265	0.04418 ± 0.01280	0.03733 ± 0.00358	0.04418 ± 0.01280	0.01109	0.12506	0.0887	−0.57404	−0.56995	−0.68199
**RNA4**	29	187	0.03282 ± 0.01034	0.02700 ± 0.00126	0.03282 ± 0.01034	0.00110	0.12596	0.00874	−0.68872	−0.73525	−0.85120
**RNA5**	25	246	0.05183 ± 0.01678	0.05552 ± 0.00318	0.05183 ± 0.01678	0.01551	0.10042	0.1548	0.28459	0.10779	0.19384

n: number of sequences; S: number of polymorphic (segregating) sites; θ*_W_*: Watterson’s Theta; π: nucleotide diversity; Hd: haplotype diversity; Pi_a_: number of nonsynonymous substitutions; Pi_s_: number of synonymous substitutions; Fu and Li’s D*: the D test that accounts for the number of mutations among alleles; Fu and Li’s F*: the *F* test that accounts for the number of segregating sites. All Tajima’s *D* and Fu and Li’s tests were not significant (*p* > 0.10).

**Table 3:** Genetic differentiation among phylogroups of RNA3.

Population I	Population II	*K*_s_*	*p*-Value	Z*	*p*-Value	Snn	*p*-Value	*F_ST_*
Clade ORF3-I	Clade ORF3-II	2.17071	0.0070 **	2.31398	0.0000 ***	1.00000	0.0013 **	0.76152
Clade ORF3-I	Clade ORF3-III	2.86276	0.0000 ***	5.42576	0.0000 ***	1.00000	0.0000 ***	0.75412
Clade ORF3-II	Clade ORF3-III	2.98920	0.0000 ***	5.51200	0.0000 ***	1.00000	0.0010 **	0.61905

** 0.001 < *p* < 0.01, *** *p* < 0.001, indicating statistical significance

## Data Availability

All sequence data have been submitted to GenBank with the accession numbers PQ438405 to PQ438536.

## Supplementary Materials

The following supporting information can be downloaded at https://www.mdpi.com/article/10.3390/microorganisms13030633/s1: Table S1: List of collected samples and GenBank accession numbers of the isolates sequenced in this study. Table S2: Summary of studied genome regions: lengths and identity percentages. Figure S1: An exemplary 1% agarose gel electrophoresis analysis of ASaV detection using specific primers S55-579F and S55-920R targeting RNA3. Figure S2: An exemplary 1% agarose gel electrophoresis analysis of ORF1 N- and C-proximal RNA1 PCR products. Figure S3: An exemplary 1% agarose gel electrophoresis analysis of the near full-length RNA2 PCR products. Figure S4: An exemplary 1% agarose gel electrophoresis analysis of the near full-length RNA3 PCR products. Figure S5: An exemplary 1% agarose gel electrophoresis analysis of the near full-length RNA4 PCR products. Figure S6: An exemplary 1% agarose gel electrophoresis analysis of the near full-length RNA5 PCR product. Figure S7: The phylogenetic tree of ASaV isolates based on the nt sequences of ORF 4. Figure S8: The phylogenetic tree of ASaV isolates based on the deduced amino acid ORF1 N-proximal.
