# Supplementary material for: Evolutionary Dynamics and Population Genetics of Ash Shoestring-Associated Virus in a European-Wide Survey
Source: Microorganisms. 2025 Mar 11;13(3):633. doi: 10.3390/microorganisms13030633 (PMC11945195; doi:10.3390/microorganisms13030633)
Supplement: Supplementary file 1 [file microorganisms-13-00633-s001.zip › Legends of Supplementary Figures S1-S6.pdf]

Legends of Supplementary Figures S1 – S6

Figure S1. An exemplary 1% agarose gel electrophoresis analysis of ASaV detection using specific primers S55-579F, and S55-920R targeting RNA3, Gaskin et al., 2021, PCR products: 1–14 = E56811, E56812, E56814, E56818, E58008, E58013, E58023, E58602, E62015, E62017, E62018, E62037, E62053, E62073. nc = negative control. Arrows indicate the size of individual bands of the standard. M: 1Kb Gene Ruler DNA Ladder (Thermo Scientific™ SM0311)

Figure S2. An exemplary 1% agarose gel electrophoresis analysis of ORF1 N–proximal RNA1 PCR products: line 1–8 = E62017, E62033, E62907, E63064, E63065, E63069, E63071, E63073, and ORF1 C–proximal RNA1 PCR products: 9–16 = E62017, E62033, E62907, E63064, E63065, E63069, E63071, E63073. nc = negative control (E62020). Arrows indicate the size of individual bands of the standard. M: 1Kb Gene Ruler DNA Ladder (Thermo Scientific™ SM0311).

Figure S3. An exemplary 1% agarose gel electrophoresis analysis of near the full-length RNA2 PCR products: 1–9= E62659, E62779, E62900, E62902, E62907, E63065, E63069, E63071, E63073. nc= negative control (E62020). Arrows indicate the size of individual bands of the standard. M: 1Kb Gene Ruler DNA Ladder (Thermo Scientific™ SM0311).

Figure S4. An exemplary 1% agarose gel electrophoresis analysis of near the full-length RNA3 PCR products: 1–7= E58008, E58051, E58602, E56811, E56814, E58023, E58013. nc = negative control (E62020). Arrows indicate the size of individual bands of the standard. M: 1Kb Gene Ruler DNA Ladder (Thermo Scientific™ SM0311).

Figure S5. An exemplary 1% agarose gel electrophoresis analysis of near the full-length RNA4 PCR products: 1–12= E62015, E62017, E62018, E62033, E62037, E62053, E62073, E62659, E62779, E62898, E62900, E62902. nc= negative control (E62020). Arrows indicate the size of individual bands of the standard. M: 1Kb Gene Ruler DNA Ladder (Thermo Scientific™ SM0311)

Figure S6. An exemplary 1% agarose gel electrophoresis analysis of near the full-length RNA5 PCR products: 1–7= E62015, E62033, E62037, E62053, E62073, E62900, E62902. nc= negative control (E62020). Arrows indicate the size of individual bands of the standard. M: 1Kb Gene Ruler DNA Ladder (Thermo Scientific™ SM0311).
