# Supplementary material for: Evolutionary Dynamics and Population Genetics of Ash Shoestring-Associated Virus in a European-Wide Survey
Source: Microorganisms. 2025 Mar 11;13(3):633. doi: 10.3390/microorganisms13030633 (PMC11945195; doi:10.3390/microorganisms13030633)
Supplement: Supplementary file 1 [file microorganisms-13-00633-s001.zip › Supplementary Figure S7.pptx]

## Slide 1
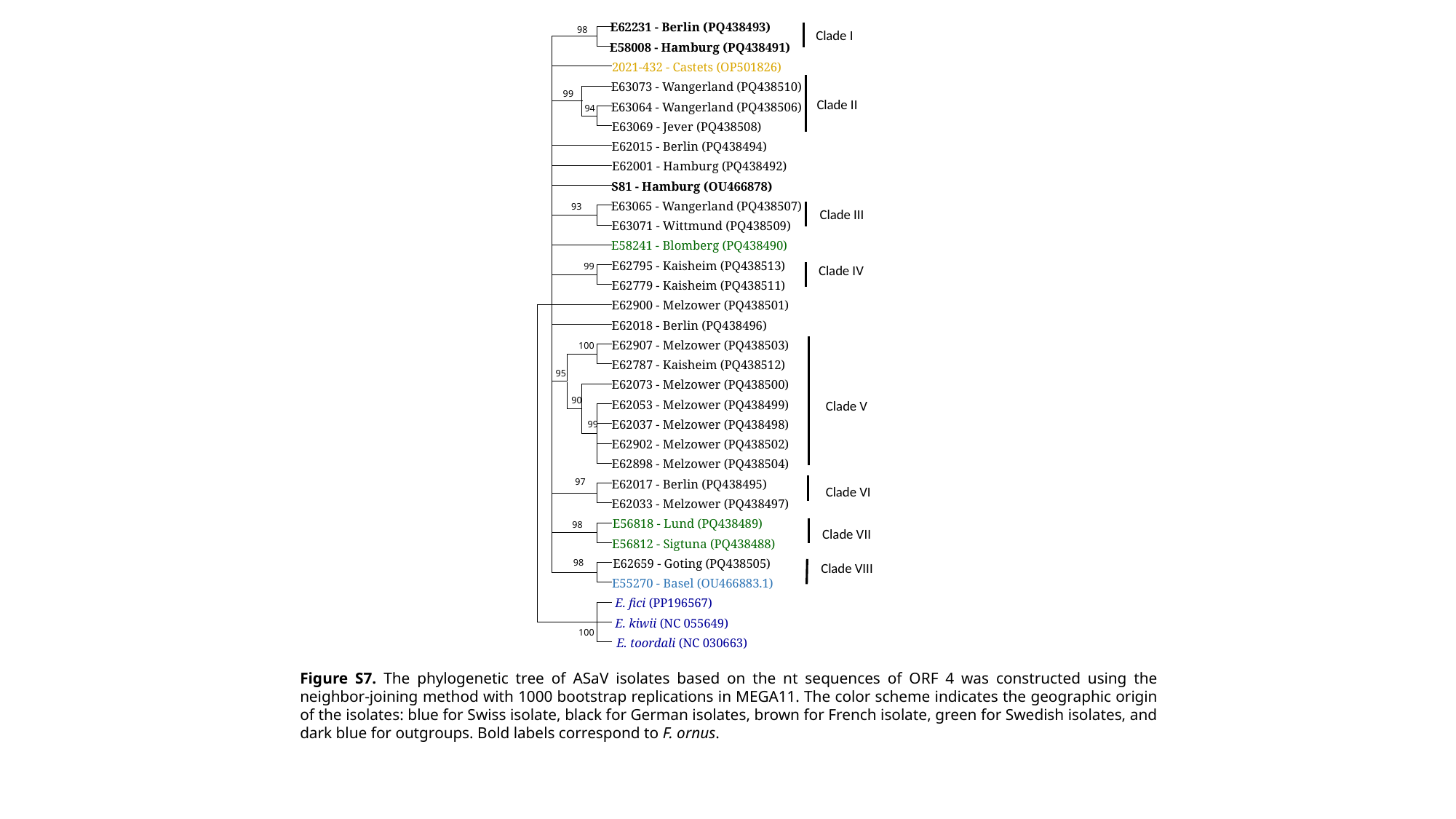

E62231 - Berlin (PQ438493)
98
 E58008 - Hamburg (PQ438491)
 2021-432 - Castets (OP501826)
 E63073 - Wangerland (PQ438510)
99
 E63064 - Wangerland (PQ438506)
94
 E63069 - Jever (PQ438508)
 E62015 - Berlin (PQ438494)
 E62001 - Hamburg (PQ438492)
 S81 - Hamburg (OU466878)
 E63065 - Wangerland (PQ438507)
93
 E63071 - Wittmund (PQ438509)
 E58241 - Blomberg (PQ438490)
 E62795 - Kaisheim (PQ438513)
99
 E62779 - Kaisheim (PQ438511)
 E62900 - Melzower (PQ438501)
 E62018 - Berlin (PQ438496)
 E62907 - Melzower (PQ438503)
100
 E62787 - Kaisheim (PQ438512)
95
 E62073 - Melzower (PQ438500)
90
 E62053 - Melzower (PQ438499)
 E62037 - Melzower (PQ438498)
99
 E62902 - Melzower (PQ438502)
 E62898 - Melzower (PQ438504)
97
 E62017 - Berlin (PQ438495)
 E62033 - Melzower (PQ438497)
 E56818 - Lund (PQ438489)
98
 E56812 - Sigtuna (PQ438488)
 E62659 - Goting (PQ438505)
98
 E55270 - Basel (OU466883.1)
 E. fici (PP196567)
 E. kiwii (NC 055649)
100
 E. toordali (NC 030663)
Clade I
Clade II
Clade III
Clade IV
Clade V
Clade VI
Clade VII
Clade VIII
Figure S7. The phylogenetic tree of ASaV isolates based on the nt sequences of ORF 4 was constructed using the neighbor-joining method with 1000 bootstrap replications in MEGA11. The color scheme indicates the geographic origin of the isolates: blue for Swiss isolate, black for German isolates, brown for French isolate, green for Swedish isolates, and dark blue for outgroups. Bold labels correspond to F. ornus.
