# Supplementary material for: Evolutionary Dynamics and Population Genetics of Ash Shoestring-Associated Virus in a European-Wide Survey
Source: Microorganisms. 2025 Mar 11;13(3):633. doi: 10.3390/microorganisms13030633 (PMC11945195; doi:10.3390/microorganisms13030633)
Supplement: Supplementary file 1 [file microorganisms-13-00633-s001.zip › Supplementary Figure S8.pptx]

## Slide 1
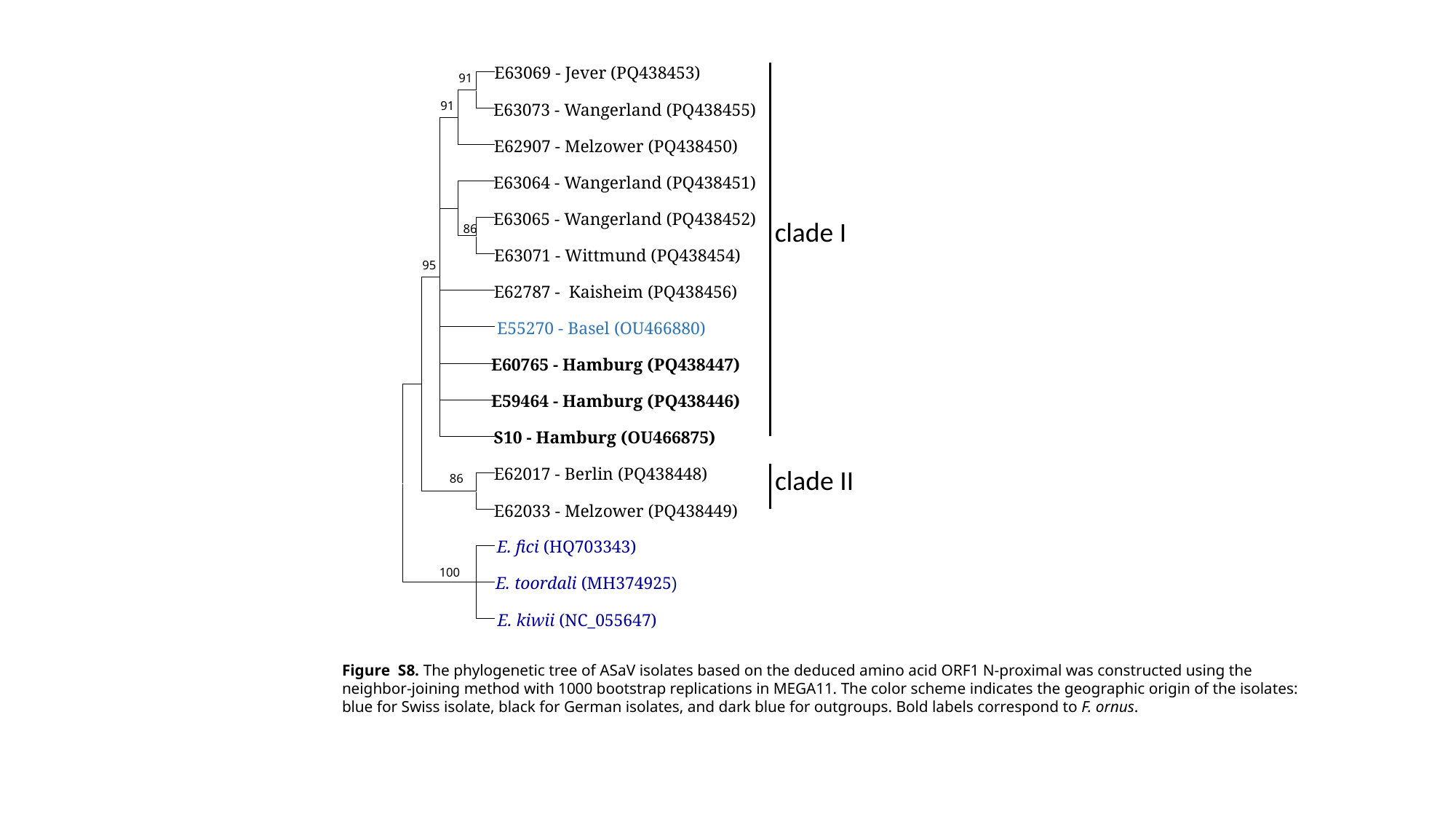

E63069 - Jever (PQ438453)
91
91
 E63073 - Wangerland (PQ438455)
 E62907 - Melzower (PQ438450)
 E63064 - Wangerland (PQ438451)
 E63065 - Wangerland (PQ438452)
86
 E63071 - Wittmund (PQ438454)
95
 E62787 - Kaisheim (PQ438456)
 E55270 - Basel (OU466880)
 E60765 - Hamburg (PQ438447)
 E59464 - Hamburg (PQ438446)
 S10 - Hamburg (OU466875)
 E62017 - Berlin (PQ438448)
86
 E62033 - Melzower (PQ438449)
 E. fici (HQ703343)
100
 E. toordali (MH374925)
 E. kiwii (NC_055647)
clade I
clade II
Figure S8. The phylogenetic tree of ASaV isolates based on the deduced amino acid ORF1 N-proximal was constructed using the neighbor-joining method with 1000 bootstrap replications in MEGA11. The color scheme indicates the geographic origin of the isolates: blue for Swiss isolate, black for German isolates, and dark blue for outgroups. Bold labels correspond to F. ornus.
