# Supplementary material for: Evolutionary Dynamics and Population Genetics of Ash Shoestring-Associated Virus in a European-Wide Survey
Source: Microorganisms. 2025 Mar 11;13(3):633. doi: 10.3390/microorganisms13030633 (PMC11945195; doi:10.3390/microorganisms13030633)
Supplement: Supplementary file 1 [file microorganisms-13-00633-s001.zip › Table S2. summary of studied genome regions lengths and identity percentages.pdf]

Table S2: Summary of studied genome regions: lengths and identity percentages

Genome region	ucleic acid length (nt)	Nucleotide identity (%)	Protein length (aa)	Amino acid identity (%)	5' UTR length (nt)	5' UTR identity (%)	3' UTR length (nt)	3' UTR identity (%)
ORF1 N-proximal	990	92.88–100	330	97.19		ns*		ns
ORF1 C-proximal	1069	87.62–99.91	356	96.81				
RNA2	2176	92.75–100	641	96.27–100	16	100	238	93–100
RNA3	1419	91.97–100	315	93.1–100	68	86–100	407	90–100
RNA4	1458	94–100	361	99.16–100	50	95.7–100	326	94–100
RNA5	1288	90–100	231	93.05–100	42	86–100	553	84–100

*not studied
