# Supplementary figures and images for: Evolutionary Dynamics and Population Genetics of Ash Shoestring-Associated Virus in a European-Wide Survey
Source: Microorganisms. 2025 Mar 11;13(3):633. doi: 10.3390/microorganisms13030633 (PMC11945195; doi:10.3390/microorganisms13030633)

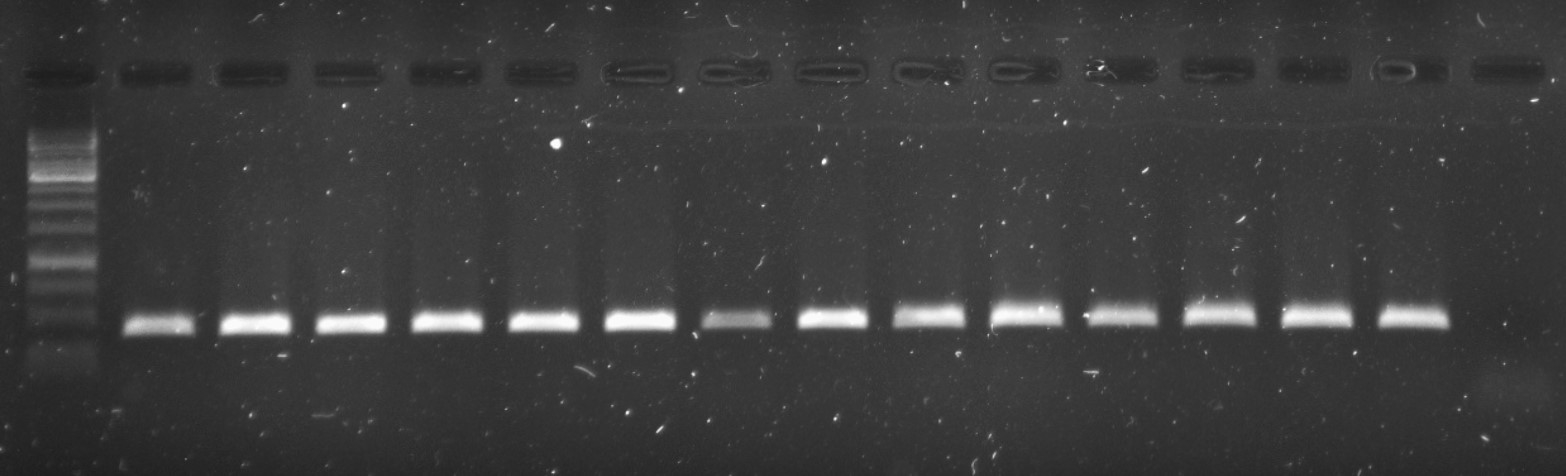

Supplement: Supplementary file 1 [file microorganisms-13-00633-s001.zip › Supplementary Figure S1.jpg]

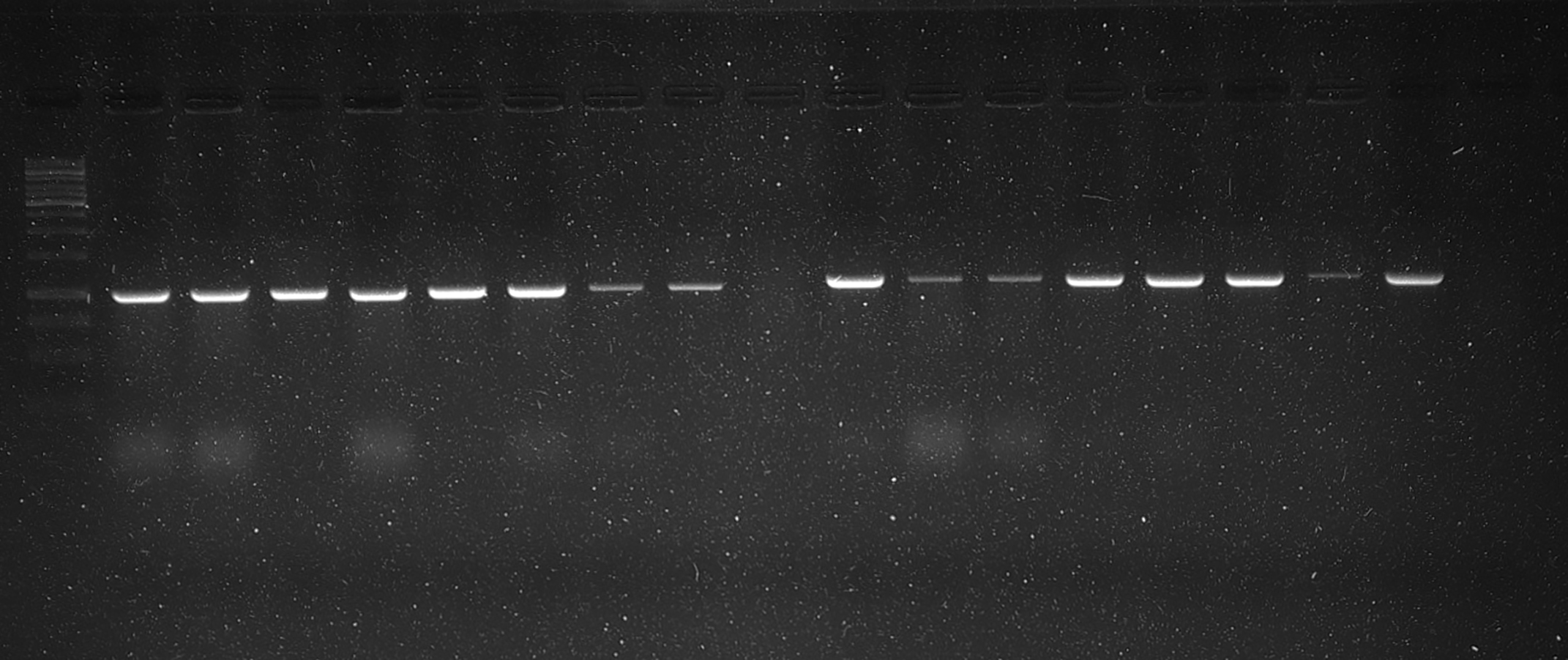

Supplement: Supplementary file 1 [file microorganisms-13-00633-s001.zip › Supplementary Figure S2.jpg]

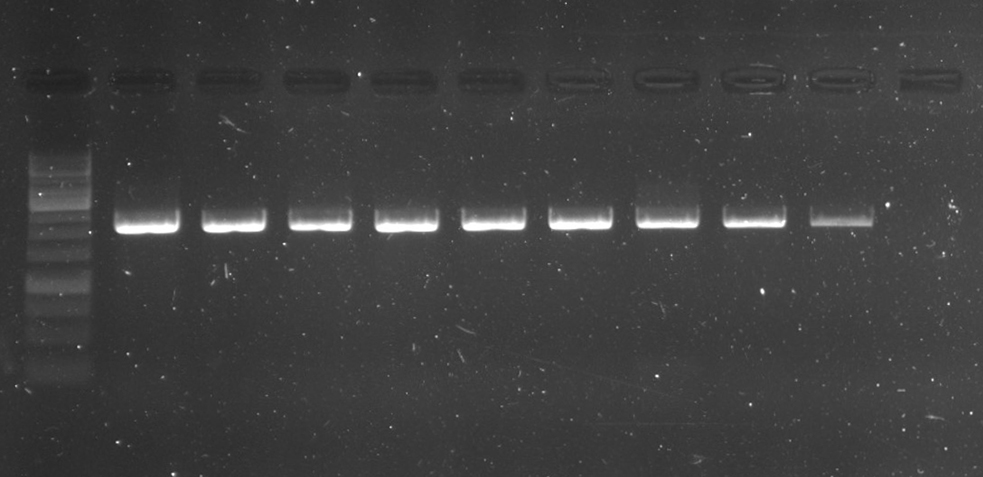

Supplement: Supplementary file 1 [file microorganisms-13-00633-s001.zip › Supplementary Figure S3.jpg]

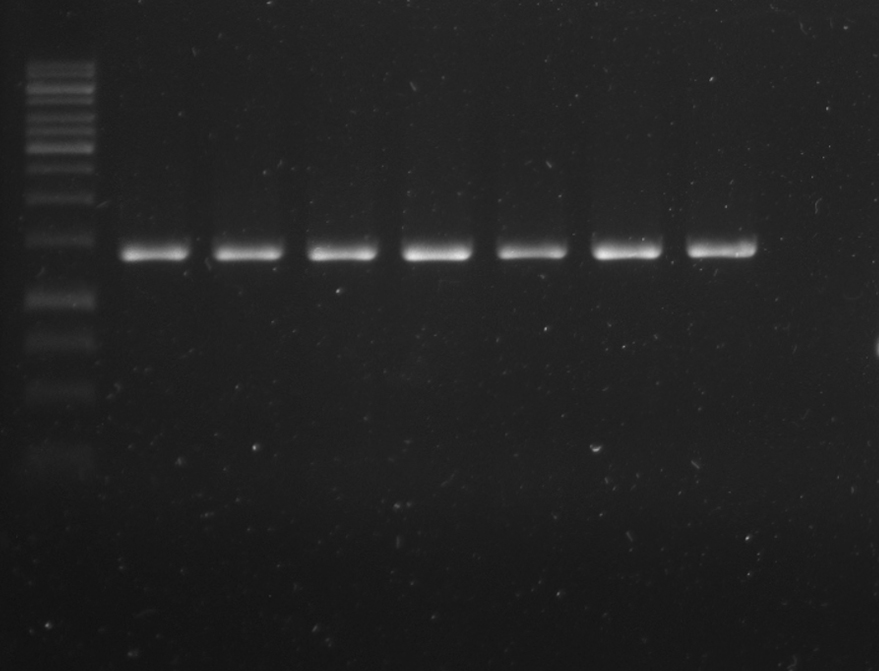

Supplement: Supplementary file 1 [file microorganisms-13-00633-s001.zip › Supplementary Figure S4.jpg]

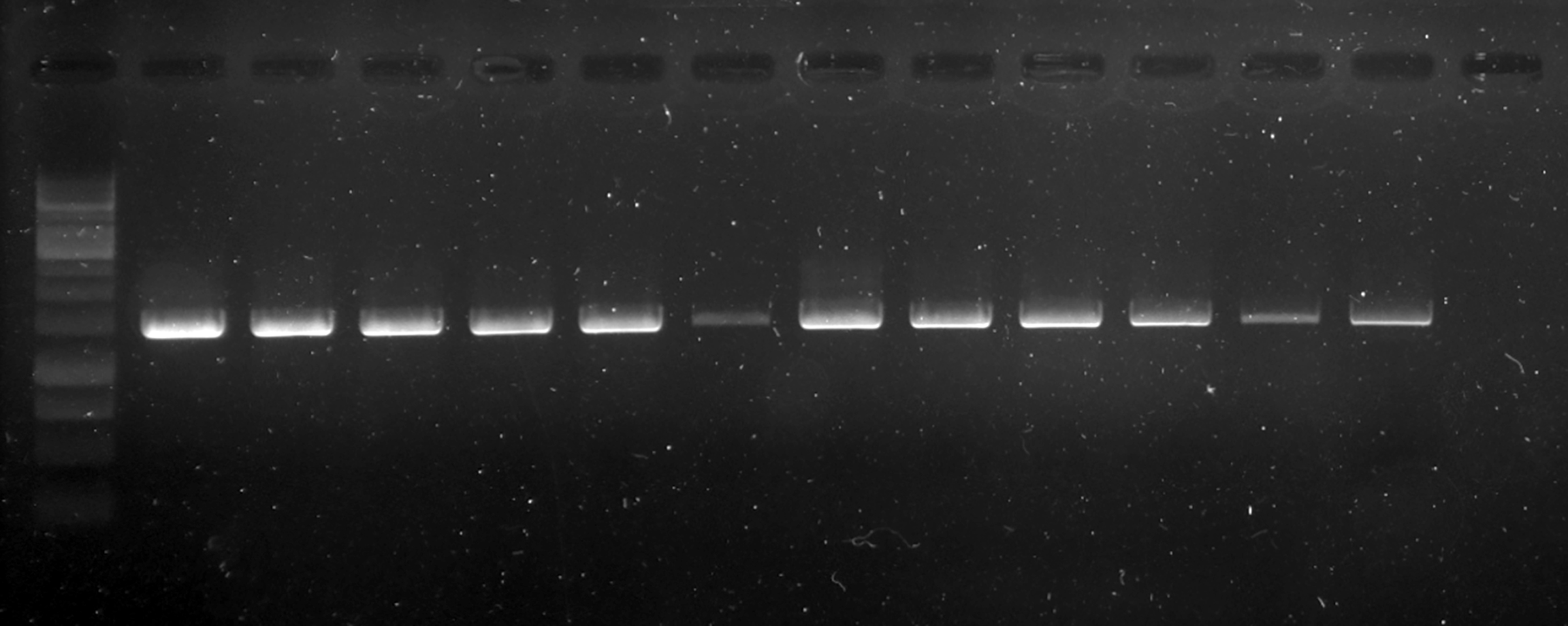

Supplement: Supplementary file 1 [file microorganisms-13-00633-s001.zip › Supplementary Figure S5.jpg]

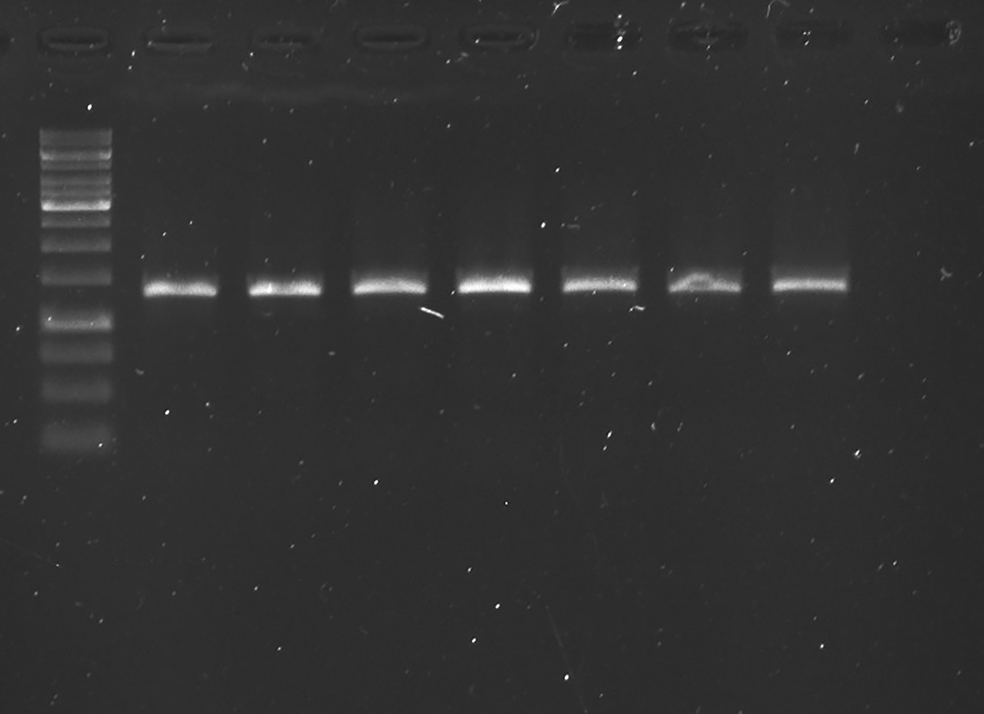

Supplement: Supplementary file 1 [file microorganisms-13-00633-s001.zip › Supplementary Figure S6.jpeg]
